# The limits of preconception care for global health

**DOI:** 10.1080/26410397.2025.2499329

**Published:** 2025-04-29

**Authors:** Miranda R. Waggoner, Michelle Pentecost

**Affiliations:** a Associate Professor, Department of Sociology, Rice University, Houston, TX, USA.; b Senior Lecturer in Anthropology and Global Health, Department of Global Health and Social Medicine, King’s College London, London, UK; c SAMRC-Wits Developmental Pathways for Health Research Unit, Faculty of Health Sciences, University of the Witwatersrand, Johannesburg, South Africa

**Keywords:** preconception health, gender equity, reproductive justice, continuum of care

## Abstract

Global health programmes aimed at reducing maternal and childhood mortality and morbidity are increasingly employing the concept of “preconception care” – an approach that the World Health Organization defines as interventions that occur before women (or couples) conceive and that address factors that could lead to poor birth outcomes. While the goal of improving maternal and child health outcomes is a vital one that is most assuredly shared by all in the global health community, the concept of preconception care is not without its limits and has significant drawbacks. From a gender rights and equity perspective, the preconception care framework has the potential to introduce harms and risks to women and people capable of getting pregnant. In this article, we summarise the key concerns about preconception care for global health in the twenty-first century. We recommend alternative frameworks that do not revolve around conception and have the potential to benefit all, including women, men, people who can get pregnant, people who do not want to get pregnant, pregnant individuals, and children.

## Résumé

Les programmes de santé dans le monde qui visent à réduire la mortalité et la morbidité maternelles et infantiles ont de plus en plus recours au concept de « soins préconceptionnels », une approche que l’Organisation mondiale de la Santé définit comme des interventions réalisées avant la conception par les femmes (ou les couples) et qui s’attaquent à des facteurs susceptibles d’entraîner des complications à la naissance. Si l’amélioration de la santé maternelle et infantile est un objectif essentiel, incontestablement partagé par l’ensemble de la communauté sanitaire mondiale, le concept de soins préconceptionnels n’est pas sans limites et présente des inconvénients majeurs. Du point de vue des droits et de l’équité entre les sexes, le cadre des soins préconceptionnels peut engendrer des préjudices et des risques pour les femmes et les personnes susceptibles de concevoir. Dans cet article, nous résumons les principales préoccupations relatives aux soins préconceptionnels pour la santé mondiale au XXI^e^ siècle. Nous recommandons d’autres cadres envisageables qui ne sont pas axés sur la conception et peuvent bénéficier à tous, y compris aux femmes, aux hommes, aux personnes susceptibles de concevoir, aux personnes qui ne veulent pas concevoir, aux personnes enceintes, et aux enfants.

## Resumen

Los programas de salud mundial destinados a reducir la morbimortalidad materno-infantil cada vez más están empleando el concepto de “atención preconcepción”, un enfoque que la Organización Mundial de la Salud define como intervenciones que ocurren antes que las mujeres (o parejas) conciben y que abordan factores que podrían causar resultados adversos del parto. Aunque mejorar los resultados de la salud materno-infantil es un objetivo vital que seguramente es compartido por todos en la comunidad de salud mundial, el concepto de atención preconcepción no está exento de límites y tiene considerables desventajas. Desde la perspectiva de derechos y equidad de género, el marco de atención preconcepción tiene el potencial de presentar daños y riesgos para las mujeres y personas con capacidad de gestar. En este artículo, resumimos las principales preocupaciones sobre la atención preconcepción para la salud mundial en el siglo XXI. Recomendamos otros marcos que no giran en torno a la concepción y que tienen el potencial de beneficiar a todas las personas: mujeres, hombres, personas con capacidad de gestar, personas que no quieren quedar embarazadas, personas embarazadas, y menores.

Global health programmes aimed at reducing maternal and childhood mortality and morbidity are increasingly employing the concept of “preconception care” – an approach that the World Health Organization^1,2^ defines as interventions that occur before women (or couples) conceive and that address factors that could lead to poor birth outcomes. Possible interventions are wide-ranging, from attending to nutritional and mental health needs to assessing environmental risks, and include biomedical, behavioural, and social components.

While public health investment in the “preconception period” is not new and has been the target of national and international health programmes since at least the 1980s,^1–3^ more recently there is renewed interest in expanding “preconception care” to include population health approaches that enfold all individuals of reproductive age. This latest momentum is based on the science of the Developmental Origins of Health and Disease (DOHaD), a field that has historically been the object of social science scrutiny for its myopic focus on the maternal body as a primary site of intervention for the health of future generations.^4^ As summarised in a 2018 *Lancet* series, preconception health is now framed as a vital period for “next generation health”^5^ that should be the target of both state surveillance and individual vigilance.^6^

This enthusiasm for the preconception focus has led to a significant increase in support for research and programmes around preconception care, with little attention paid to the possible effects of such a shift on reproductive rights and gender equity.^7^

While the goal of improving maternal and child health outcomes is a vital one that is most assuredly shared by all in the global health community, the concept of preconception care is not without its limits and has significant drawbacks. From a gender rights and equity perspective, the preconception care framework has the potential to introduce harms and risks to women and people capable of getting pregnant. In this article, we summarise the key concerns about preconception care for global health in the twenty-first century. We recommend alternative frameworks that do not revolve around conception and have the potential to benefit all, including women, men, people who can get pregnant, people who do not want to get pregnant, pregnant individuals, and children.

## The problem of language

When it is used to refer to care, the term “preconception” is confusing, deterministic, confining, unfamiliar to lay people, and difficult to translate into other languages. While the use of the term “preconception” to define a specific biological period is required for scientists doing basic research, there are several issues that arise when the term is used for population health science and global health interventions.

First, the term fosters temporal confusion.^3^ “Preconception” is vague and expansive – it can refer to interventions immediately before conception, some months before conception, or throughout the reproductive years leading up to conception. Proponents of preconception care explicitly frame it as a population health concern that should target all people of reproductive age, which introduces the question of when preconception care should begin. The notion that a focus on pre-pregnancy care should begin in adolescence risks framing all health issues for young people as secondary to and in service of their reproductive health, with attendant issues for equity. For interventions with a demonstrable evidence base, such as preconception folic acid supplementation,^8^ their efficacy has been described when used around the time of conception. That is, the critical juncture is often the *periconception* period rather than the *preconception* period. Instead of targeting all people of reproductive age as always potentially pregnant,^9^ there needs to be clarity and transparency about the importance of the timing of interventions, and this includes using clear terminology.

Second, the term also implies a durable, even deterministic, link between preconception care and outcomes, when, in fact, it is difficult to ascertain the impact of preconception care on various maternal and child health outcomes. The evidence base for many interventions before pregnancy is insufficient for the kinds of claims that are often made about the importance of individual lifestyle changes. To be sure, there is good evidence for interventions such as folic acid supplementation around the time of conception for the prevention of neural tube defects,^8^ and there are promising findings from trials assessing the effect of multidomain interventions (health, nutrition, water, and sanitation) in women who wish to conceive.^10^ Pre-pregnancy care targeted at optimising specific medical conditions also improves neonatal outcomes – for example, ensuring optimised management of diabetes prior to pregnancy reduces the risk of congenital malformations, preterm delivery, and neonatal intensive care admission.^11^ However, the bulk of the existing DOHaD evidence base is observational, and the interpretation of this evidence base has frequently been shaped by strong underlying assumptions that maternal factors around the time of pregnancy have a powerful causal role in shaping offspring outcomes.^12^

Third, because the term revolves around conception as the principal pivot point for care, it poses problems for debates around women’s reproductive access and rights. References to the term “conception” are often used to rationalise constraints on women’s lives, which both contradicts and threatens their human rights. Conception as a health endpoint (or starting point) narrows the public health focus and jeopardises comprehensive reproductive and sexual health care, including when it comes to pregnancy termination.

Finally, there are practical concerns about the terminology as well. There is an English-language specificity to the “preconception” term that guides programme development and implementation, and yet the term is not easily translatable across all languages. The term is also unfamiliar to lay English speakers.^13,14^ Additionally, while the term is commonly used in policy documents and clinical guidelines, there are multiple perspectives among preconception health researchers on what should constitute the preconception period (see Stephenson et al^5^) and the preconception population.^15^ “Preconception” means different things to different people in terms of the time interval before conception and is poorly and inconsistently defined in the academic literature.^15^ As a term, it does not adhere to the rationale that patient communication should be in plain language that is non-judgemental and respectful of patients’ personal preferences.^16^ From a primary care perspective, people may bristle at care language that is animated by conception or pregnancy when they are not seeking pregnancy. In the U.S., for example, the “every woman, every time” strategy means that any time a woman has an interaction with a health care provider, she should be asked about her reproductive intentions and receive appropriate counsel.^17^ Yet, such an approach of viewing all women as potential reproductive bodies undermines gender equity and is freighted with assumptions about women’s priorities and how they should conduct their lives.

Given the multiple problems besetting the term, the politics it channels (even if inadvertently), and the confusion and determinism that it cultivates about the relationship among interventions, timing, and outcomes, it is inadvisable for global and national policies and programmes to focus on “preconception”. Preconception is a scientific term of use for basic scientists, but when it is used in a programmatic context it fosters confusion and can be marshalled in social, cultural, and political debates that are consequential for gender equity. It must be remembered that language that is used by global health leaders is deployed within – and can contribute to – social, cultural, and policy contexts that have high stakes for gender equity.

## Gendered terminology, gendered ethic

The terminology of “preconception care” also indexes a broader ethic of reproductive control that predominantly targets women. While “preconception” sounds ungendered on the surface,^18^ and while preconception care initiatives in recent years have targeted men, it is still primarily women in society who carry pregnancies. Preconception care remains a framework that couches health and care interventions in the name of future pregnancies – pregnancies that are gestated predominantly by women.

The prohibition against sex discrimination in international human rights law requires that women be treated as individuals – not as fetal vessels – in society and in health care.* It is worth underscoring here that many women do seek and desire healthy pregnancies, of course. But not all women of reproductive age want to be pregnant, and pregnancy should not be the central organising principle for women’s health.

Notwithstanding, women have historically – and unjustly – been regularly reduced to sex-specific physiological characteristics, as in pregnancy. And the expectations and assumptions around women’s standing and promise in society have been infused with cultural notions of maternity, motherhood, and pronatalism. Indeed, the preconception care term catalogues a broader ethic of anticipatory motherhood that envisions all women of childbearing age as potential mothers and exhorts them to prepare their bodies for future pregnancies.^3,19^ This ethic is pronatalist and assumes that anticipatory motherhood is desirable. It also situates, both socially and culturally, the normative way to be a potential mother: to be a “good” mother is to anticipate future motherhood, practice preconception care, and reduce all possible risk to a future fetus.

It is an ethic, too, that ignores the various sexual and reproductive health care needs of women, such as abortion. There has long been a tendency to avoid the topic of abortion in preconception care frameworks,^3,19^ and preconception care typically operates under the assumption that pregnancies can be – and will be – planned and subsequently carried to term. Preconception care indeed implies that there is a straightforward relationship between preconception care and intentionality. And yet the notion of a “planned,” “unplanned,” or “unintended” pregnancy is fraught and difficult to measure, as people think about these concepts in a layered and nuanced way.^20^

Furthermore, not all people desire parenthood, and not all people are in relationships that have the possibility of producing a pregnancy. The expectation that all people capable of pregnancy will abide by an ethic of anticipatory motherhood is unfair, untenable, and unattainable, and it sets women up for failure.

It is unethical to offer reproductive care that averts discussion of the multiple possible outcomes of an eventual pregnancy, including termination, and that avoids recognition of the wide variability that exists when it comes to relationships and pregnancy intentionality. Care before pregnancy should not automatically default to care for anticipated reproductive futures. It should also not be assumed that pregnancies that might occur in the future will lead to a live birth or be continued at all.

## Inviting potential criminalisation and stigma

As all women of reproductive age are positioned as potential mothers, and as the ethic of anticipatory motherhood becomes a widespread expectation with preconception care, the boundaries of maternal culpability for reproductive outcomes are also expanded. Preconception care programmes give the sense that preconception care is the answer for reducing adverse birth outcomes, and this has massive and disturbing implications. Because if programmes, providers, and the public equate preconception behaviours with birth outcomes, then a woman’s preconception life can be targeted.

During pregnancy, a person’s civil liberties are already severely curtailed in many parts of the world, and pregnant people are subject to surveillance, criminalisation, and coerced interventions. In a ground-breaking study, Lynn Paltrow and Jeanne Flavin^21^ reported on hundreds of cases between 1973 and 2005 in the U.S. where pregnant women were arrested, detained, charged with a crime, or forced to undergo medical interventions (such as caesarean sections) without their consent, with Black women disproportionately suffering from this surveillance and criminalisation regime (See also Amnesty International,^22^ James,^23^ and Roberts^24^). These injurious measures have been taken against women when they act in ways that ostensibly harm the fetus, even when the evidence base for understanding the mechanisms for actual or potential harm is lacking.^21,25,26^

Here, we bring attention to how preconception care invites additional surveillance of women who are *not* pregnant, which could be dangerous and have very real consequences in specific geographic locations experiencing the rise of reproductive care restrictions, anti-abortion legislation, and hyper-surveillance and criminalisation of women’s reproductive bodies – where, for instance, women are being jailed for miscarriage and stillbirth.^27^ Preconception care is couched in terms of future pregnancy health and suggests that what one does in the preconception period is part of one’s risk profile for a number of reproductive outcomes. As such, we must fear that the trend of depriving women of their reproductive autonomy in the name of fetal health will be extended to their non-pregnant reproductive lives, leading to worsened inequities in health that are both gendered and racialised.

Moreover, a preconception framework can exacerbate ableist definitions of positive birth outcomes, as it fuels the myth that a perfect pregnancy can be planned for and achieved.^3,28,29^ It can also intensify stigma when adverse birth outcomes do occur. The notion of preconception harm and risk has long been gendered,^30,31^ and blame for reproductive outcomes is already often unduly placed on women and mothers.^32,33^ When global health focuses on who *could be* pregnant and what they should be doing to ensure healthy future reproduction, women can be labelled as bad mothers before they ever even conceive, opening them up to social, moral, and legal scrutiny.

## Preconception care’s programmatic implications

A focus on preconception care in global and national policy has several implications for resource allocation and where public health efforts are targeted.

First, women’s health services that are not defined in terms of pregnancy can be deprioritised, shifting attention and resources away from broad reproductive care.^3^ Information and awareness of risk and protective factors related to pregnancy should be provided to women and men as part of a holistic sexual and reproductive health approach. Women should have access to the full range of reproductive services throughout their lives, including termination options. It is notable that recent scholarship has called for integrating preconception and contraception care,^34^ and some initiatives, such as the One Key Question® screening tool (i.e. asking someone whether they would like to become pregnant in the next year), place intentionality and pregnancy planning squarely within a preconception care framework.^35,36^ While these are innovative programmatic ideas, they centre a rhetoric of choice (when “choice” might not be accessible to all) and continue to cue women to think about future reproduction as the organising component of their care. Preconception care, even when offered as part of contraceptive care or pregnancy planning, and even to the extent that it aligns with reproductive justice principles,^37^ still relies on conversations about conception. In short, we argue that concern for the health and lives of women and girls and people capable of conceiving should not pivot on the possibility of their becoming pregnant in the future.

Second, the specialist needs of particular population groups may be overlooked. Preconception care has largely functioned as a heteronormative and binary framework. Gay and lesbian couples seeking parenthood have mostly been left out of preconception care interventions and materials, as have trans individuals. Other population health frameworks have the potential to be more inclusive while eschewing gendered stereotypes and a gendered ethic.

Third, a programmatic implication of preconception care is that attention is focused on individualised interventions rather than structural ones. While there are certainly important ongoing discussions in global health about broader factors that contribute to reproductive risk and maternal and child health disparities, preconception care has by and large been rolled out as an individualised health approach. By focusing on individualised reproductive care and life plans, intellectual and programmatic resources are steered away from bigger social factors, such as poverty, racism, or misogyny, that put the health of many people – including that of pregnant and potentially pregnant people – at risk. Is it ethical to pursue an individual-level approach when it siphons attention and resources from some of the factors that matter the most to people’s health, including that of the next generation?

## Thinking beyond preconception care for global health

As we have argued, it is dangerous to define health care needs of people solely in relation to their capacity to become pregnant. The preconception care framework is at odds with a wider shift in acknowledgement and understanding of the broad spectrum of people’s reproductive and sexual health needs.^38^ Non-judgemental attitudes towards women, men, and all people should be an explicit component of reproductive care, inclusive of care that occurs among non-pregnant people who have the capacity for future pregnancy. People who do not seek out or who refuse preconception care should not be characterised or judged as irresponsible, uncaring, or as in any way jeopardising the health of any future pregnancy or future child. And they should not be targeted or surveilled for any behaviours or lifestyles that may increase risk in a future pregnancy.

We propose that global health imperatives should be guided not by a focus on preconception care but rather by the contributions made by scholars and activists advancing a framework of reproductive justice, defined as “the right to have children, to not have children, and to parent the children we have in safe and healthy environments”.^39,40^ In keeping with the aim of “sexual and reproductive health rights for all”,^41^ this approach understands and underscores reproductive rights as fundamental human rights and espouses the principles of upholding sexual and reproductive health autonomy, non-discrimination, and non-violence. While health interventions that focus on preparation for pregnancy might have a positive impact on reproductive autonomy, the preconception care framework does not encompass all of the values and aspects of a framework that privileges reproductive and sexual health equity^38^ and might even be detrimental to these values and worsen existing inequities.

For example, when care prior to conception is delivered, it should be in a manner that encompasses the range of possible outcomes. For those opting into care expressly focused on preparation for pregnancy, the primary aim should be discussion of the physical, mental, and social health and well-being of those contemplating pregnancy and parenthood. Such care should not betray assumptions or judgement surrounding the patient’s desires, and it should recognise the lived experiences of people, especially those of minoritised women or non-pregnant people living with medical conditions.^42^ Integrated health care interventions should be available to everyone, and such care should safeguard and respect the wishes and the dignity of the people seeking it, taking care not to stigmatise or position individuals as blameworthy for any possible future reproductive outcomes.

A continuum of care approach is preferable to preconception care, as it is applicable to all life course strategies and focuses on delivering patient-centred and integrated health care that caters to the patient’s needs over time. All of the goals and aims attached to the current focus on preconception care are easily enfolded in a continuum of care framework that considers each individual’s specific needs at each stage of the life course, without defining such care based on the prospect of future conception. A continuum of care approach thus achieves the specified goals of the preconception care focus, but without the attendant problems outlined here and their potential impacts on gender equity and human rights. Global health programmes must protect sexual and reproductive health autonomy, do no harm, promote inclusivity, and prevent discrimination.^41^ The current population health push for preconception care does not champion these goals, and its limits for global health should be recognised.

## Footnotes

* We thank Eszter Kismödi for this point.

## Provenance statement

This article was not commissioned and went through external peer review. While the Chief Executive of the *SRHM* organisation discussed the development of this article with the authors, all editorial decisions were made independently by the SRHM journal team following peer review.
